# Protective effects of *Radix Pseudostellariae* polysaccharides against exercise-induced oxidative stress in male rats

**DOI:** 10.3892/etm.2013.942

**Published:** 2013-01-31

**Authors:** ZICHAO CHEN, SHANSHAN LI, XIAOQIN WANG, CHUAN LONG ZHANG

**Affiliations:** 1 Department of Physical Education, Sichuan University, Chengdu, Sichuan 610064;; 2 West China Second University Hospital, Sichuan University, Chengdu, Sichuan 610041;; 3 Luoyang Institute of Science and Technology, Luoyang, Henan 471023, P.R. China

**Keywords:** *Radix Pseudostellariae* polysaccharides, swimming exercise, oxidative stress, rats

## Abstract

The main purpose of this study was to examine the effect of *Radix Pseudostellariae* polysaccharides (RPPs) against swimming exercise-induced oxidative stress in male rats. A total of 40 male Wistar rats were randomized into four groups: the control (C), low-dose RPP supplementation (LRS), medium-dose RPP supplementation (MRS) and high-dose RPP supplementation (HRS) groups. The control group received saline solution and the supplementation groups received different doses of RPPs (100, 200 and 400 mg/kg body weight, respectively). The animals were medicated orally and daily for 28 days. On day 28, the rats were made to swim until exhausted. The exhaustive swimming time and various biochemical parameters, including blood lactate, hemoglobin, catalase (CAT), superoxide dismutase (SOD), glutathione peroxidase (GSH-Px) and malondialdehyde (MDA), were measured. The results showed that RPP supplementation elevates the exercise tolerance and decreases the blood lactate level of rats following exhaustive swimming exercise. RPP supplementation augments the levels of hemoglobin and anti-oxidant enzymes (CAT, SOD and GSH-Px), and effectively decreases the MDA content of the skeletal muscle of rats, which suggests that RPP supplementation has a protective effect against exercise-induced oxidative stress.

## Introduction

During the past few decades, a series of studies have addressed the association of strenuous physical exercise with increased oxygen uptake and generation of reactive oxygen species (ROS) in various mammals (1–4). In general, the body has adequate antioxidant reserves to cope with the production of ROS under physiological conditions. The antioxidant system consists of antioxidant vitamins, glutathione and thiols, and antioxidant enzymes. Each of these antioxidants plays a unique role within the cell and complements the others functionally (5). However, when ROS levels exceed the normal physiological coping range during strenuous physical exercise, the accumulation of ROS and a reduction in antioxidant status may result. This scenario increases oxidative stress and leads to modifications of lipid and protein structures that consequently compromise the cellular functions in tissue (6,7). To increase the body’s antioxidant potential and to decrease levels of oxidative stress, it has been recommended that individuals increase their intake of dietary antioxidants. Dietary antioxidants interact with endogenous antioxidants to form a cooperative antioxidant network (8).

*Radix Pseudostellariae*, the root of *Pseudostellaria heterophylla* (Miq.) Pax. known as ‘Tongshen’ or ‘Taizishen’, has a long history of medicinal use in China. As a traditional Chinese medicine, it is frequently used to treat disease, in particular as a lung and spleen tonic (9,10). A number of studies concerning *Radix Pseudostellariae,* including its chemical components and relevant pharmacological properties, have been performed. *Radix Pseudostellariae* polysaccharides (RPPs) have displayed clear anti-infectious, anti-inflammatory, hypoglycemic, hypolipidemic and immunomodulating activities (11–14). Studies have also shown that RPPs exhibit strong antioxidant activities (15), which suggests that they are beneficial in counteracting exercise-induced oxidative stress. However, the effects of RPPs on exercise-induced oxidative stress have not been investigated thus far. Therefore, in the present study, we investigated the effects of RPP supplementation against the exercise-induced oxidative stress of forced swimming in male rats.

## Materials and methods

### Materials and chemicals

The dried *Radix Pseudostellariae* (native to Shandong herbal medicines planting base, China) was purchased from Tongjitang Herb Shop (Chengdu, China). The authenticity of the plant was confirmed by Dr MF Li, a botanist at Sichuan University (Chengdu, China), and a voucher specimen was deposited in the Herbarium of Sichuan University. The dried *Radix Pseudostellariae* was ground with an electric mixer prior to extraction. The assay kits for blood lactate, hemoglobin, catalase (CAT), superoxide dismutase (SOD), glutathione peroxidase (GSH-Px) and malondialdehyde (MDA) were purchased from Jianchen Bioengineering Institute (Nanjing, China). Other chemicals and biochemicals were of analytical grade and were purchased from Sigma Chemical Co. (St. Louis, MO, USA) and Changsheng Pharmaceutical Co. (Chengdu, China) unless otherwise indicated.

### Experiment animals

Healthy male Wistar rats with an average mass of 225–250 g were obtained from Sichuan Research Animal Center (Chengdu, China). A standard pellet diet and water were provided *ad libitum*. The animals were housed in a temperature-controlled room at 21–23°C and maintained on a 12 h light : 12 h dark cycle. All animals received humane care according to the guidelines of the Guidebook for the Care and Use of Laboratory Animals (16). The study protocol was approved by the Animal Research Ethics Committee at Sichuan University (Chengdu, China).

### Preparation of RPPs

The preparation of RPPs was carried out according to the literature (17,18). In brief, the dried *Radix Pseudostellariae* was ground into powder. The powder (400 g) was extracted three times by refluxing with 80% ethanol (1 liter) at 90°C for 2–3 h each time. After filtration, the dregs were extracted again three times with water (1.5 liter) at 90°C for 2–3 h each time. The extracted solution was condensed to 400 ml and deproteinated using the Sevag method. The solution was then added to absolute ethanol until the ethanol concentration was 80% and kept overnight, followed by filtration. The precipitate was dissolved with water (100 ml) and then absolute ethanol was added until the ethanol concentration was 80%, the solution was filtrated and this method was repeated once again. The precipitate was washed with 95% ethanol, absolute ethanol and acetone by turns, and then dried at 50°C.

### Experimental protocol

A total of 40 healthy male Wistar rats were randomized into four equal groups based on body weight following one week where rats acclimated to the new environment: the control (C), low-dose RPP supplementation (LRS), medium-dose RPP supplementation (MRS) and high-dose RPP supplementation (HRS) groups. The C group received saline solution and the supplementation groups received different doses of RPPs (100, 200 and 400 mg/kg body weight, respectively). The treatments were administered orally and daily for 28 days.

Following the final supplementation with RPPs or saline solution, the rats were allowed to rest for 30 min. The rats were then removed for the exhaustive swimming exercise. The details of this apparatus have been reported previously (19). A acrylic plastic pool (90×60×60 cm) was filled with water to a depth of 40 cm and maintained at 28±1°C. The rats were forced to swim in the water, and the endurance was defined as the time active swimming was maintained until the animal submerged in the water without movement. To diminish stress, all rats had been accustomed to swimming with repeated short-term swimming sessions for a week prior to experimentation.

### Analysis of biochemical parameters

At the end of the swimming test, the rats were anesthetized with pentobarbital sodium (5 mg/100 g body weight, i.p.). Blood was obtained from the orbital sinus for lactate and hemoglobin level measurements. Hindlimb skeletal muscle was rapidly removed and homogenized immediately in ice-cold 10% KCl solution (10 ml/g of tissue) using a teflon/glass homogenizer. The suspension was centrifuged at 671 × g at 4°C for 10 min and the clear supernatant was used for CAT, SOD, GSH-Px and MDA level measurements. All biochemical parameters were determined using commercial kits following the manufacturer’s recommended instructions.

### Statistical analysis

The data are expressed as the mean ± SD. Statistical comparisons were compared by one-way analysis of variance (ANOVA). P<0.05 was considered to indicate a statistically significant result.

## Results and Discussion

### Effects of RPP supplementation on the exhaustive swimming times of rats

**Figure 1:**
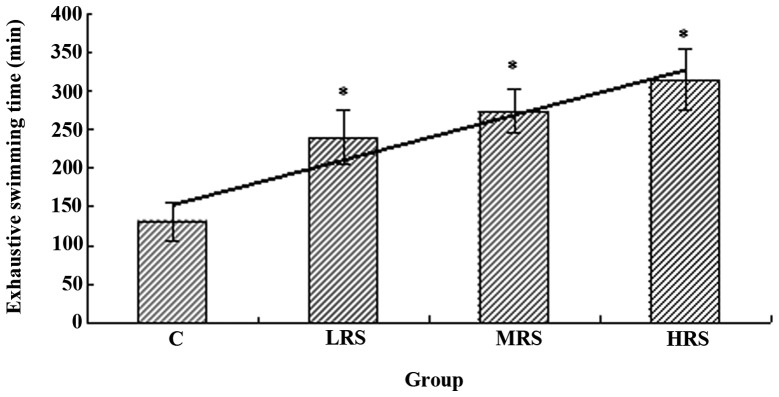
Effects of RPP supplementation on the exhaustive swimming times of rats. Data are presented as the mean ± SD of ten rats per group. ^*^P<0.05, compared with the control (C) group. RPP, *Radix Pseudostellariae* polysaccharide; LRS, low-dose RPP supplementation; MRS, medium-dose RPP supplementation; HRS, high-dose RPP supplementation.

Exhaustive swimming was selected as a model of physical exercise since muscle trauma caused by other types of physical exercise, including prolonged running on a treadmill, exercise stimulated by electric shock and plyometric contractions, may be avoided (20,21). As shown in Fig. 1, exhaustive swimming times in all the RPP supplementation groups were significantly longer compared with that of the C group (P<0.05). These results indicate that RPP supplementation is able to elevate exercise endurance.

### Effects of RPP supplementation on the blood lactate levels of rats

**Figure 2:**
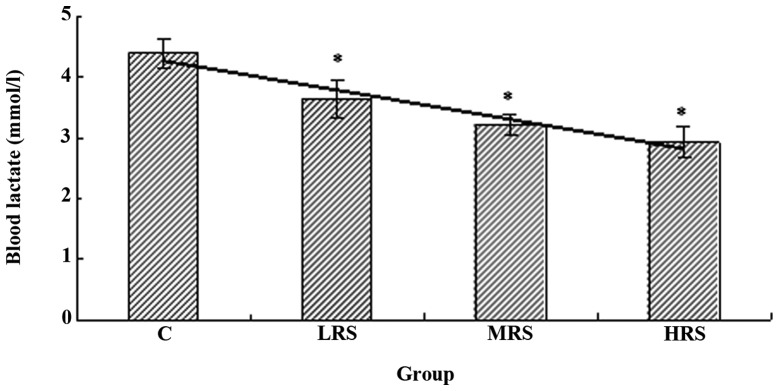
Effects of RPP supplementation on the blood lactate level of rats. Data are presented as the mean ± SD of ten rats per group. ^*^P<0.05, compared with the control (C) group. RPP, *Radix Pseudostellariae* polysaccharide; LRS, low-dose RPP supplementation; MRS, medium-dose RPP supplementation; HRS, high-dose RPP supplementation.

Lactate serves as an energy source in highly oxidative tissues. Numerous organs, including the liver and heart, and tissues such as skeletal muscle, aid the removal of lactate from the blood, but intense exercise increases lactate production (22). As shown in Fig. 2, the blood lactate level in each of the RPP supplementation groups was significantly lower compared with that of the C group (P<0.05). These results indicated that RPP supplementation effectively attenuates the increase of blood lactate, which may be responsible for the improvement in exercise endurance.

### Effects of RPP supplementation on the hemoglobin levels of rats

**Figure 3:**
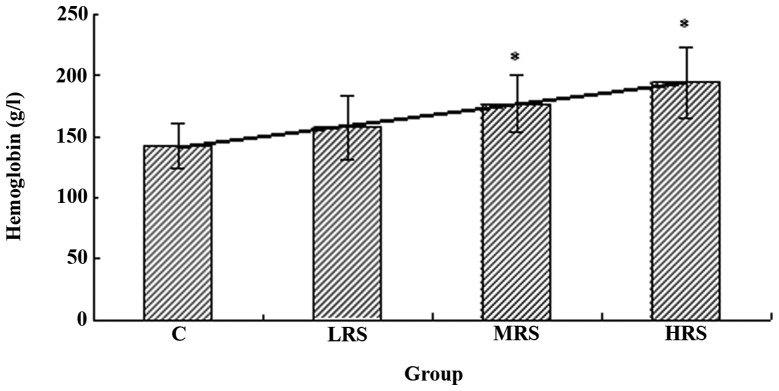
Effects of RPP supplementation on the hemoglobin level of rats. Data are presented as the mean ± SD of ten rats per group. ^*^P<0.05, compared with the control (C) group. RPP, *Radix Pseudostellariae* polysaccharide; LRS, low-dose RPP supplementation; MRS, medium-dose RPP supplementation; HRS, high-dose RPP supplementation.

Hemoglobin is the main component of erythrocytes. The improvement of cardiopulmonary function and increase of oxygen supply to tissues caused by an increase in hemoglobin levels are commonly stated to be major factors that increase endurance capacity (23). As shown in Fig. 3, the hemoglobin levels in the MRS and HRS groups were significantly higher compared with that of the C group (P<0.05). Although the hemoglobin level in the LRS group was also increased, no significant difference was observed (P>0.05). These results indicated that RPP supplementation may influence the supply of oxygen to tissues by hemoglobin, and may contribute to the improvement in exercise endurance.

### Effects of RPP supplementation on the MDA content of rat skeletal muscle

**Figure 4:**
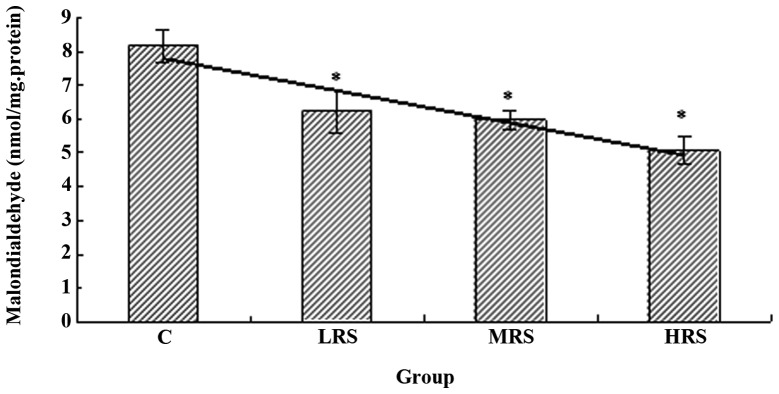
Effects of RPP supplementation on the MDA content of skeletal muscle in rats. Data are presented as the mean ± SD of ten rats per group. ^*^P<0.05, compared with the control (C) group. RPP, *Radix Pseudostellariae* polysaccharide; LRS, low-dose RPP supplementation; MRS, medium-dose RPP supplementation; HRS, high-dose RPP supplementation.

Lipid peroxidation represents oxidative tissue damage caused by hydrogen peroxide, superoxide anions and hydroxyl radicals, resulting in structural alteration of the membrane, release of cell and organelle content and loss of essential fatty acids with formation of cytosolic aldehyde and peroxide products (24). MDA is a secondary product generated during the oxidation of polyunsaturated fatty acids, which has been frequently measured as an indicator of lipid peroxidation and oxidative stress *in vivo* (25). Numerous studies have observed that strenuous physical exercise induces increases in the MDA concentration in tissues (24,26,27). As shown in Fig. 4, the MDA content in all three RPP supplementation groups was significantly lower compared with that of the C group (P<0.05). These results indicate that RPP supplementation effectively reduces lipid peroxidation.

### Effects of RPP supplementation on the antioxidant enzyme content of rat skeletal muscle

CAT, SOD and GSH-Px are regarded as the first line of defense by the antioxidant enzyme system against ROS generated during exhaustive exercise (28). SOD catalyzes the dismutation of superoxide into oxygen and hydrogen peroxide. GSH-Px is a selenoenzyme which catalyzes the reduction of hydroperoxides at the expense of reduced glutathione. CAT is a primary antioxidant defense component that catalyzes the decomposition of hydrogen peroxide to water, sharing this function with GSH-Px (29). As shown in Table I, the CAT contents in the MRS and HRS groups were significantly higher compared with that of the C group (P<0.05). Although the CAT content in the LRS group was also increased, no significant difference was observed (P>0.05). The SOD and GSH-Px contents in the RPP supplementation groups were significantly higher compared with that of the C group (P<0.05). These results indicate that RPP supplementation upregulated antioxidant enzymes to protect against oxidative stress-induced injury during exhaustive exercise.

From the present findings, we conclude that RPP supplementation elevates the exercise tolerance and decreases the blood lactate level of rats following exhaustive swimming exercise. RPP supplementation augments the levels of hemoglobin and antioxidant enzymes and effectively decreases the MDA content in the skeletal muscle, which suggests that RPP supplementation possesses protective effects against swimming-induced oxidative stress. Our data relates to rats, therefore future studies using different subjects, possibly of different sporting backgrounds, are required to extend these findings.

## Figures and Tables

**Table I:** Effects of RPP supplementation on the antioxidant enzyme content of skeletal muscle in rats.

Group	CAT (U/mg protein)	SOD (U/mg protein)	GSH-Px (U/mg protein)
C	3.16±0.32	101.31±11.42	6.57±1.38
LRS	3.33±0.41	148.53±10.78^a^	9.86±1.85^a^
MRS	4.15±0.39^a^	162.87±13.46^a^	12.39±1.16^a^
HRS	4.48±0.46^a^	169.52±12.24^a^	16.34±1.72^a^

Data are the mean ± SD of ten rats per group.

a P<0.05, compared with the control (C) group. RPP, *Radix Pseudostellariae* polysaccharides; CAT, catalase; SOD, superoxide dismutase; GSH-Px, glutathione peroxidase; LRS, low-dose RRP supplementation; MRS, medium-dose RRP supplementation; HRS, high-dose RRP supplementation.
